# *Apterous A* modulates wing size, bristle formation and patterning in *Nilaparvata lugens*

**DOI:** 10.1038/srep10526

**Published:** 2015-05-21

**Authors:** Fangzhou Liu, Kaiyin Li, Jie Li, Dingbang Hu, Jing Zhao, Yueping He, Yulan Zou, Yanni Feng, Hongxia Hua

**Affiliations:** 1 Hubei Insect Resources Utilization and Sustainable Pest Management Key Laboratory, College of Plant Science and Technology, Huazhong Agricultural University, Wuhan, China; 2 College of Agronomy and Plant Protection, Qingdao Agricultural University, Qingdao, China; 3 College of Life Science and Technology, Huazhong Agricultural University, Wuhan, China

## Abstract

*Apterous A* (*apA*), a member of the LIM-homeobox gene family, plays a critical role in the development of wing. The achaete-scute Complex (AS-C) encodes basic helix-loop-helix (bHLH) transcription factors and functions in bristle development. In the present study, we cloned *apA* (*NlapA*) and an achaete-scute homologue (*NlASH*) from *N. lugens*. Levels of *NlapA* and *NlASH* were higher in nymphs than adults, with particularly high expression in the thorax of nymphs. *NlapA* expressed more highly in nymphs of the macropterous strain (MS) than those of the brachypterous strain (BS) at 2^nd^ and 4^th^ instar. Knockdown of *NlapA* and *NlASH in vivo* generated similar phenotypic defects in the wing (loss-of-bristles, twisted or erect wing). Silencing of *NlapA* in nymphs of MS led to decreased wing size in adults. Moreover, depletion of *NlapA* suppressed expression of *NlDl*, *Nlsal*, *Nlser*, *Nlvg* and *Nlwg*, both in MS and BS, but induced differential responses of *Nlubx* and *Nlnotch* expression between MS and BS. Notably, expression of *NlASH* was regulated by *NlapA*. These results collectively indicate that *NlapA* is an upstream modulator of wing size, bristle formation and patterning. Further studies on DNA-protein and protein-protein interactions are required to elucidate *NlapA*-mediated regulation of wing development.

The acquisition of the ability to fly is one of the most important events in insect evolution, and undoubtedly a major reason for their evolutionary success. The molecular mechanisms underlying wing developmental processes have attracted extensive research attention. Genes related to wing development of holometabolous *Drosophila melanogaster* have been comprehensively characterized to date. Before identification of wing patterning genes in ants and aphids, Abouheif and Wray (2002) and Brisson *et al.* (2010) deduced the presumptive gene network involved in wing patterning from *D. melanogaster* based on previous literatures^1^,^2^. While limited information is available on genes related to wing development in hemimetabolous insects. All wing patterning genes have been identified from hemimetabolous *Acyrthosiphon pisum*, and the expression levels of 11 of these genes at embryogenesis and across nymphal instars between winged and wingless populations compared^2^. However, few reports have focused on the functions of wing patterning genes of hemimetabolous insects.

The *Drosophila Apterous* gene (*ap*) is a member of the LIM-homeobox gene family, which encodes a transcription factor and contains a DNA-binding homeodomain plus two cysteine-rich LIM domains^3^,^4^. *Ap* plays a crucial role in wing development. Restricted expression and activity of *ap* are responsible for development of the dorsal and ventral compartments of the *Drosophila* wing imaginal disc^5^,^6^,^7^. In *ap* mutants, the wing is lost^6^,^7^, possibly because of loss of expression of genes regulated by *ap*, such as *Serrate*, *Fringe* and *integrins*^8^,^9^. While several studies have characterized *ap* in holometabolous insects, its role in wing development of hemimetabolous insects remains unknown.

Genes of the *achaete/scute* (*ASH*) gene family encode basic helix-loop-helix (bHLH) transcription factors and are widely conserved throughout the animal kingdom^10^. The achaete-scute complex (AS-C) induces the formation of external sensory organs that function in mechano- and chemo-sensory reception. In *Drosophila*, *ac/sc* is expressed in the proneural clusters. Some of the cells are selected to develop into sensory organ precursors, and subsequently, mature bristles^11^,^12^. The *ac/sc* double mutant leads to loss of the majority of bristles, while ectopic expression of *ac* and/or *sc* induces extra bristles in flies^13^,^14^,^15^,^16^. A 26 bp deletion within the *Bm-ASH2* (*ac/sc* homologue of *Bombyx mori*) promoter is closely linked to the scaleless mutation, and leads to loss of *Bm*-*ASH*2 expression and the scaleless-wing phenotype, indicating that *Bm*-*ASH*2 plays a critical role in scale formation in *B. mori*^17^. Selection of the sensory organ precursor is associated with high levels of *ac/sc* expression maintained by regulators, such as Sens, Pannier, Chip, dLMO, Ap, Wingless and Shaggy. These activators or suppressors act in concert to regulate expression of *ASH*^18^,^19^,^20^,^21^,^22^,^23^,^24^,^25^. At present, no reports on *ASH* identification and characterization in hemimetabolous insects are available in the literature.

The brown planthopper (BPH), *Nilaparvata lugens* (Stål) (Hemiptera: Delphacidae), is one of the most destructive insect pests of rice. BPH has two winged morphs, specifically, long-winged (migratory) and short-winged (non-migratory), leading to difficulty in its control. The effects of environmental and genetic factors on wing development and wing dimorphism of BPH are well documented^26^, but the combinatorial actions of genes responsible for wing development and morph differentiation remain unclear. The ecdysone receptor is known to control wing morphogenesis and melanization in *N. lugens*^27^. Disruption of Krüppel-homolog 1 expression via RNAi in BPH causes stunted wing development and malformation of both male and female external genitalia^28^. Downregulation of BPH *Distal-less* in 3^rd^-instar larvae leads to disrupted development of the leg, and that of *NlDll* in 5^th^- instar larvae to abnormal wing formation^29^.

In the present study, we cloned the homologue of *ap* from *N. lugens*, investigated the expression patterns in the brachypterous strain (BS) and macropterous strain (MS), and determined its role in wing morph differentiation and development. The *ac/sc* homologue from *N. lugens* responsible for bristle formation of wing was additionally cloned and characterized. Expression levels of downstream target genes of *ap*, such as *wingless* (*wg*), *Notch*, *ultrabithorax* (*ubx*), *vestigial* (*vg*), *spalt major* (*sal*), *Delta* (*Dl*) and *serrate* (*ser*) of *N. lugens*, were quantified after knockdown of *ap*. To our knowledge, this is the first report on *ap* and *ac/sc* cloning and characterization in hemimetabolous *N. lugens*.

## Results

### Characterization of *NlapA* cDNA

Full-length cDNA of *ap* of *N. lugens* was isolated from wing pads of nymphs using the rapid amplification of cDNA ends (RACE) method. Clustal W analysis revealed that the *ap* homologue belongs to the *apA* class (*NlapA*) (Supplementary Fig. S1). Full-length *NlapA* cDNA (GenBank accession No: KC978728) was determined as 3004 bp, with a 5’ untranslated region (UTR) of 57 bp, 3’ UTR of 1678 bp, and an open reading frame (ORF) of 1269 bp (Supplementary Fig. S2). *NlapA* encoded 422 amino acid residues with a predicted molecular weight of 46.6 kDa and pI of 8.93. The deduced amino acid sequence was predicted to contain two cysteine/histidine-rich domains known as ‘LIM domains’, located amino terminal to a homeodomain (Supplementary Fig. S2).

Phylogenetic analysis using the coding region of *NlapA* was conducted for determining the evolutionary pattern among insects. NlapA was most closely related to apA of *Tribolium castaneum*, whereby both appeared to originate from a common ancestor, and most distantly related to ap of *Apis floreal* (Supplementary Fig. S3).

Comparison of the deduced amino acid sequence of *NlapA* with apA of *A. pisum* (XP_001946004.2), *T. castaneum* (NP_001139341.1), *B. mori* (BAK19079.1) and *Danaus plexippus* (EHJ74086.1) revealed the highest sequence identity (75.5%) with apA of *T. castaneum*, followed by *A. pisum* (57.1%), *B. mori* (50.6%), and *D. plexippus* (50.1%).

### Characterization of *NlASH* cDNA

cDNA of an *ASH* homologue of *N. lugens* (*NlASH*, GenBank accession No: KM244736) was amplified from total RNA of wing pads of nymphs. EXPASY analysis indicated that *NlASH* includes a full-length coding sequence. The start and stop codons are shown in Supplementary Fig. S4. *NlASH* encoded 233 residues with a predicted molecular weight of 23.9 kDa and pI of 10.09. One basic helix-loop-helix (bHLH) motif was predicted from the deduced amino acid sequence (Supplementary Fig. S4).

Phylogenetic analysis was performed using the coding region of *NlASH* to determine the evolutionary pattern among insects. *NlASH* was most closely related to *ASH* of *Apis mellifera* and *Nasonia vitripennis* (all appeared to originate from a common ancestor) and most distantly related to *ASH1* of *Culex pipiens* (Supplementary Fig. S5).

Comparison of the deduced amino acid sequence of *NlASH* with ASH of *A. mellifera* (NW_001252982), *N. vitripennis* (XP_003426998.1), and *T. castaneum* (NP_001034537.1), SC of *D. melanogaster* (NP _476803.1) and AC of *D. melanogaster* (NP_476824.1) revealed the highest sequence identity (47.4%) with AS-C of *N. vitripennis*, followed by AS-C of *T. castaneum* (44.1%), AS-C of A. *mellifera* (43.3%), SC of *D. melanogaster* (39.9%), and AC of *D. melanogaster* (36.1%).

### Expression patterns of *NlapA* and *NlASH*

**Figure 1:**
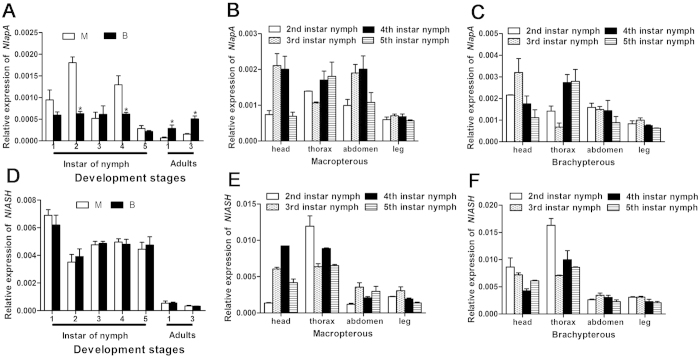
The expression pattern of *NlapA* and *NlASH*. (**A**) The relative expression pattern of *NlapA* at different developmental stages of MS and BS; (**B**) The tissue-specific expression pattern of *NlapA* in nymphs of MS; (**C**) The tissue-specific expression pattern of *NlapA* in nymphs of BS; (**D**) The relative expression pattern of *NlASH* at different developmental stages of MS and BS; (**E**) The tissue-specific expression pattern of *NlASH* in nymphs of MS; (**F**) The tissue-specific expression pattern of *NlASH* in nymphs of BS. BPH *actin1* was used as reference control. The expression level was quantified relative to the value of *actin1.* The average expression level was based on three biological replicates. Error bars indicate standard errors. Bars labeled with asterisk differed significantly between the BPHs of MS and BS, as determined using one way ANOVA (*P* < 0.05).

*NlapA* was expressed from 1^st^ instar nymphs to 3 days after emergence. The *NlapA* level was significantly higher in nymphs of MS than those of BS at 2^nd^ and 4^th^ instar, and higher in adults of BS than those of MS at 1 and 3 days after emergence (Fig. 1A). *NlapA* was expressed in the head, thorax, abdomen and leg of 2^nd^, 3^rd^, 4^th^ and 5^th^ instar nymphs in a tissue- and development-specific manner. In MS, high expression was detected in the head at 3^rd^ and 4^th^ instar, thorax at 4^th^ and 5^th^ instar, and abdomen at 3^rd^ and 4^th^ instar (Fig. 1B). In BS, high expression was detected in the head at 3^rd^ instar, and thorax at 4^th^ and 5^th^ instar (Fig. 1C).

*NlASH* was also expressed from 1^st^ instar nymphs to 3 days after emergence. There was no significant difference in *NlASH* expression level between insects of MS and BS at each development stage (Fig. 1D). Expression of *NlASH* during the larval stages was significantly higher than that at the adult stage, and peaked at 1^st^ instar (Fig. 1D). *NlASH* was expressed in the head, thorax, abdomen and leg of 2^nd^, 3^rd^, 4^th^ and 5^th^ instar nymphs, with highest expression in thorax. Highest expression was detected in the thorax of 3^rd^ instar nymphs (*P* < 0.05) (Fig. 1E,F).

### Effects of dsRNA microinjection on gene expression of *NlapA* and *NlASH*

**Figure 2:**
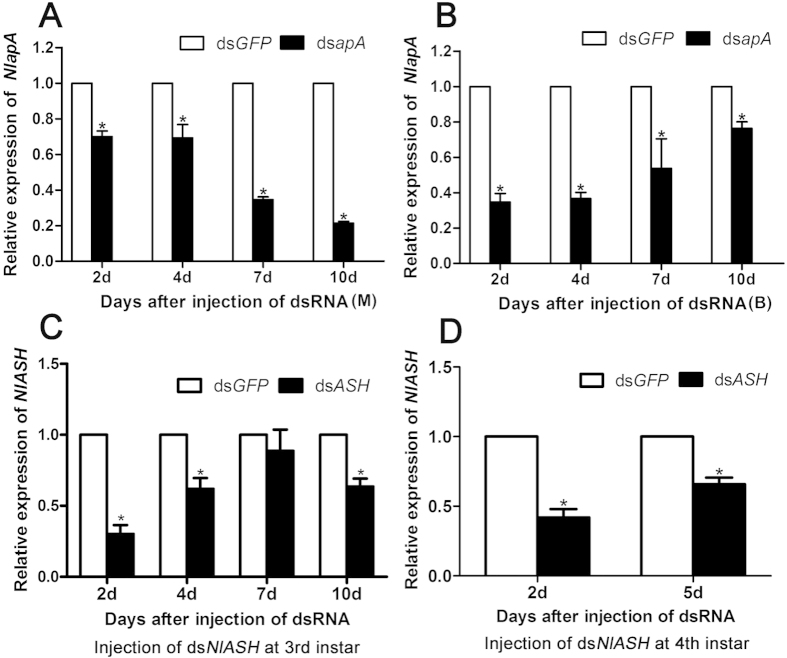
The relative expression levels of *NlapA* and *NlASH* after injection of dsRNA at 3rd-instar (400ng per nymph). (**A**) The relative expression levels of *NlapA* in BPHs of MS after injection of ds*NlapA*; (**B**) The relative expression levels of *NlapA* in BPHs of BS after injection of ds*NlapA*; (**C**) The relative expression levels of *NlASH* after injection of ds*NlASH* into 3rd- instar nymphs; (**D**) The relative expression levels of *NlASH* after injection of ds*NlASH* into 4th-instar nymphs. Error bars indicate standard errors. The expression level was quantified relative to the value of the nymphs which were injected with ds*GFP*. Bars labeled with asterisk differed significantly between the treatments on the same day, as determined using one way ANOVA (*P* < 0.05). BPH *actin1* was used as reference control. The average expression level was based on three biological replicates.

Both in MS and BS, injection of ds*NlapA* into 3^rd^ instar nymphs significantly suppressed the expression of endogenous *NlapA* mRNA as early as 2 d after treatment, and at all four sampling points. From the second to 10^th^ day after injection, transcript levels of *NlapA* were significantly decreased by 30.8%–78.7% and 23.6%–65.3% in MS and BS, respectively (*P* < 0.05, one-way ANOVA) (Fig. 2A,B). When nymphs at 3rd-instar were injected with ds*NlASH*, *NlASH* transcript levels were significantly decreased by 11.4%–69.8% from the second to 10^th^ day after injection (*P* < 0.05, one-way ANOVA) (Fig. 2C). Treatment of nymphs at 4^th^ instar with ds*NlASH* led to a significant decrease in the transcript level by 34.2%–58.1% from days 2 to 5 after injection (*P* < 0.05, Fig. 2D).

### Effects of knockdown of *NlapA* and *NlASH* on wing development *in vivo*

**Figure 3:**
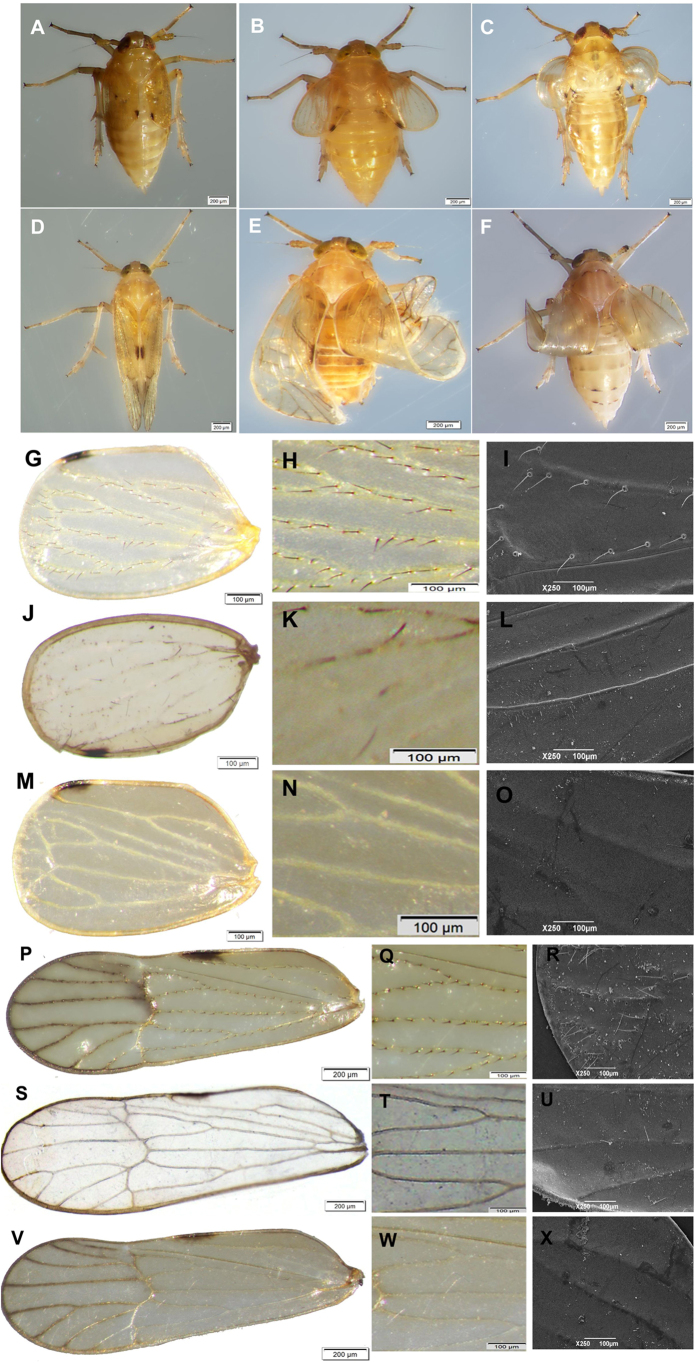
The wing phenotypes of BPHs adults emerged from nymphs injected with ds*NlapA*or ds*NlASH* (400ng per nymph) at 3rd-instar. (**A**) (**D**) short-winged and long-winged adults developed from nymphs treated with ds*GFP*; (**B**) (**E**) short-winged and long-winged adults developed from nymphs treated with ds*NlapA*; (**C**) (**F**) short-winged and long-winged adults developed from nymphs treated with ds*NlASH*; (**G**) (**H**) (**I**) forewing of short-winged adults developed from nymphs treated with ds*GFP* (magnified 60 times, 100 times and 250 times respectively); (**J**) (**K**) (**L**) forewing of short-winged adults developed from nymphs treated with ds*NlapA* (magnified 60 times, 100 times and 250 times respectively); (**M**) (**N**) (**O**) forewing of short-winged adults developed from nymphs treated with ds*NlASH* (magnified 60 times, 100 times and 250 times respectively); (**P**) (**Q**) (**R**) forewing of long-winged adults developed from nymphs treated with ds*GFP* (magnified 60 times, 100 times and 250 times respectively); (**S**) (**T**) (**U**) forewing of long-winged adults developed from nymphs treated with ds*NlapA* (magnified 60 times, 100 times and 250 times respectively); (**V**) (**W**) (**X**) forewing of long-winged adults developed from nymphs treated with ds*NlASH* (magnified 60 times, 100 times and 250 times respectively).

The specific function of *NlapA* in wing development of *N. lugens* was investigated using *in vivo* knockdown. dsRNAs were injected into 3^rd^ instar nymphs, and when adults emerged, wing phenotypic defects were observed. The results clearly indicated a deleterious effect on wing development of *N. lugens*. Compared to ds*GFP* injection, adults in both MS and BS groups injected with ds*NlapA* displayed loss-of-bristle forewings (MS: 74.5 ± 3.9%; BS: 70.5 ± 4.9%. Fig. 3J,K,L,S,T,U), and twisted or erect wings (MS: 54.8 ± 1.7%; BS: 26.3 ± 3.2%; Fig. 3B,E).

Injection of ds*NlASH* into 3^rd^ instar nymphs led to significant suppression of *NlASH*, as expected. Adults treated with ds*NlASH* exhibited similar phenotypic defects as those injected with ds*NlapA,* with loss-of-bristle forewings (89.4 ± 3.3%, Fig. 3M,N,O,V,W,X) and twisted or erect wings (28.1 ± 2.8%, Fig. 3C,F).

### Effects of knockdown of *NlapA* on wing size *in vivo*

**Figure 4:**
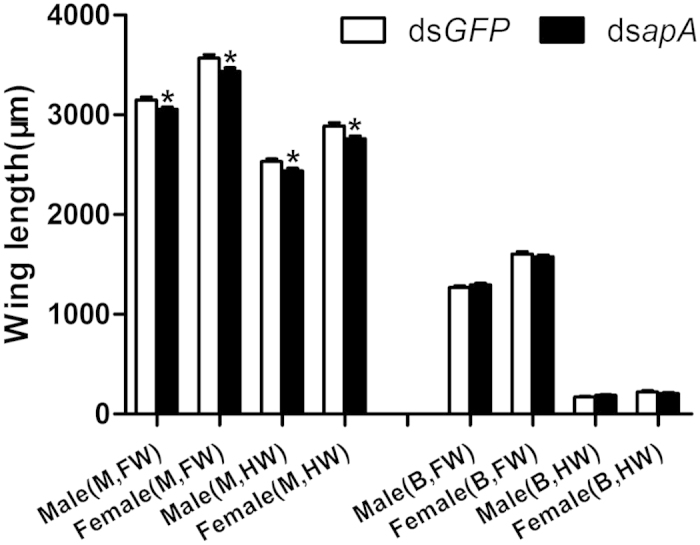
The wing length of BPHs adults treated with ds*NlapA* and ds*GFP* (400ng per nymph) at 3rd-instar. Error bars indicate standard errors. Bars labeled with asterisk differed significantly between the treatments, as determined using one way ANOVA (*P* < 0.05). M: macropterous adults; B: brachypterous adults; FW: forewing; HW: hindwing.

Wing sizes of adults emerging from nymphs injected with ds*NlapA* were measured to establish the role of *NlapA* in wing morph differentiation. The lengths of the fore- and hindwing from the base to tip of adults that emerged from 3^rd^-instar nymphs of MS injected with ds*NlapA* were significantly shorter, compared to those of adults treated with ds*GFP*, both in the female and male groups (Fig. 4). In contrast, the wing size of adults emerging from 3^rd^-instar nymphs of BS was not affected by ds*NlapA* injection. This finding indicates that *NlapA* is involved in wing morph differentiation to some extent.

### *NlapA* regulation of *NlASH* expression

**Figure 5:**
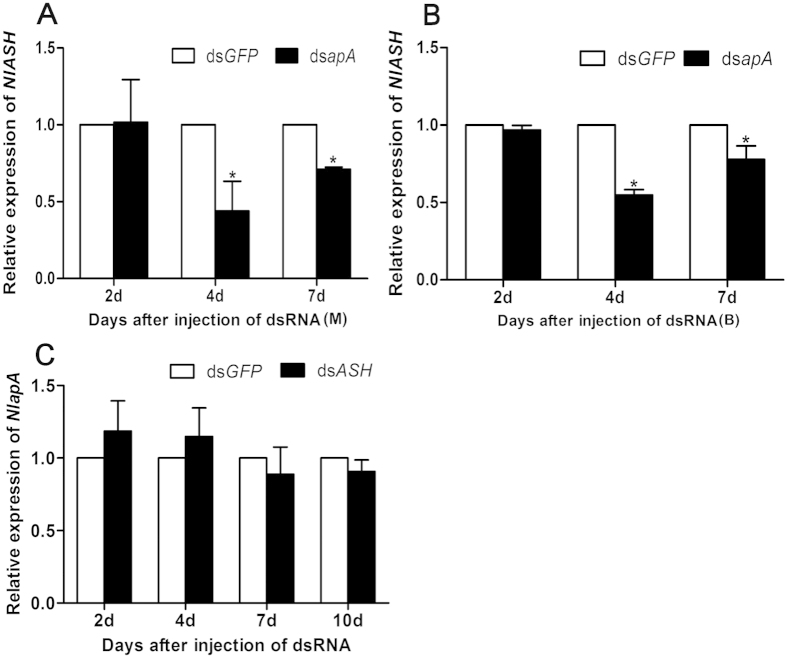
The relative expression levels of *NlapA* and *NlASH* after injection of dsRNA (400ng per nymph). (**A**) The relative expression levels of *NlASH* in MS after injection of ds*NlapA* at 3rd-instar; (**B**) The relative expression levels of *NlASH* in BS after injection of ds*NlapA* at 3rd-instar; (**C**) The relative expression levels of *NlapA* after injection of ds*NlASH* at 3rd-instar. Error bars indicate standard errors. Error bars indicate standard errors. The expression level was quantified relative to the value of the nymphs which were injected with ds*GFP*. Bars labeled with asterisk differed significantly between the treatments on the same day, as determined using one way ANOVA (*P* < 0.05). BPH *actin1* was used as reference control. The average expression level was based on three biological replicates.

The effect of *in vivo* knockdown of *NlapA* on expression of *NlASH* was investigated using qRT-PCR. Expression of *NlASH* decreased significantly from 4 to 7 d after ds*NlapA* injection in both MS and BS (Fig. 5A,B). However, knockdown of *NlASH* had no effect on expression of *NlapA* (Fig. 5C). Our results indicate that *NlapA* is an upstream regulator of *NlASH*, which is responsible for the development of forewing bristles, and normal folding and stretching of wing.

### Effect of dsRNA injection on *N. lugens* survival

**Figure 6:**
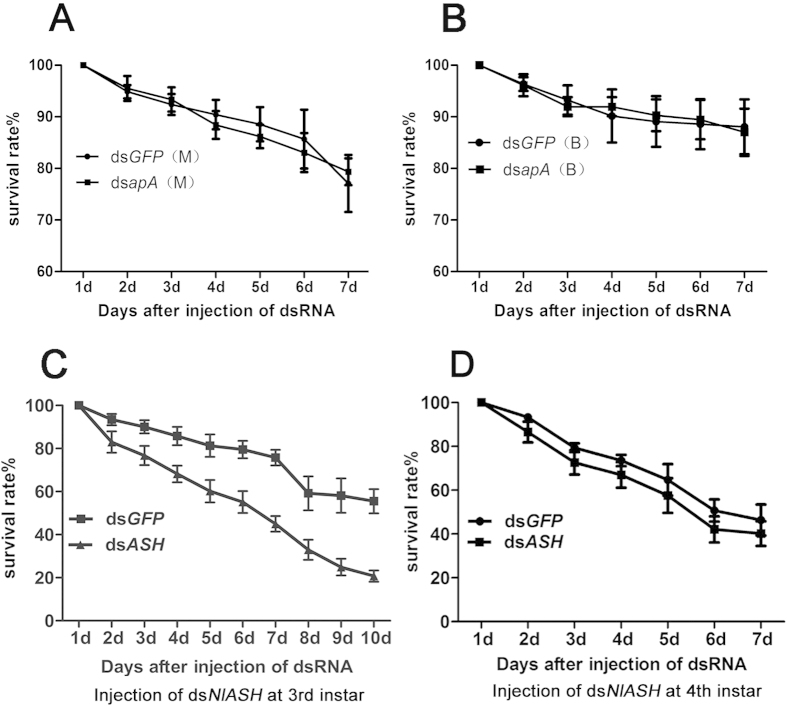
Survival response of N. lugens to ds*NlapA* and ds*NlASH* injection at 3rd-instar or 4th-instar. (**A**) Survival response of BPH*s* of MS to ds*NlapA* injection at 3rd-instar; (**B**) Survival response of BPH*s* of BS to ds*NlapA* injection at 3rd-instar; (**C**) Survival response of BPH*s* to ds*NlASH* injection at 3rd-instar; (**D**) Survival response of BPH*s* to ds*NlASH* injection at 4th-instar. ds*GFP* injection was served as negative control. Each n = 3 groups of 45 nymphs. Survival responses to the ds*GFP* and ds*NlASH* were analyzed using Repeated Measure ANOVA: (**A**).F1,4 = 0.015; *P* = 0.908; (**B**). F1,4 = 0.002; *P* = 0.965; (**C**). F1,4 = 16.164; *P* = 0.016; (**D**). F1,4 = 1.021; *P* = 0.369. M: macropterous strain; B: brachypterous strain.

We observed no significant differences in survival rates between BPHs treated with ds*GFP* and ds*NlapA* at 2, 4, 7, and 10 d (Fig. 6A,B), indicating that ds*NlapA* has no lethal effects on *N. lugens*. Following treatment of nymphs at 3^rd^ instar with ds*NlASH*, the survival rate was significantly decreased, compared to that in nymphs treated with ds*GFP* (*P* < 0.05, Fig. 6C), implying that ds*NlASH* causes lethality in this case. However, when nymphs at 4^th^ instar were treated with ds*NlASH*, the survival rate was not affected (*P* < 0.05, Fig. 6D).

### Effects of *in vivo* knockdown of *NlapA* on expression of other genes in the wing-patterning network

**Figure 7:**
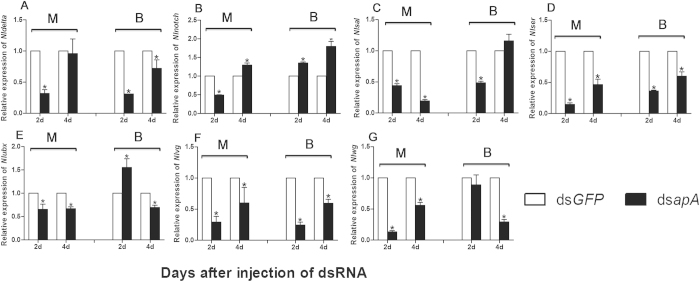
The effects of *in vivo* knockdown of *NlapA* on expression levels of *Nldl*, *Nlnotch*,*Nlsal*,*Nlser*, *Nlubx*,*Nlvg and Nlwg*. The expression level was quantified relative to the value of the insects which were injected with ds*GFP*. All error bars indicate the SE of the mean, as determined from three independent replicates. Bars labeled asterisk differed significantly between the treatments on the same day, as determined using one way ANOVA (*P* < 0.05). BPH *actin1* was used as reference control. M: macropterous strain; B: brachypterous strain.

Within the deduced wing-patterning gene network of *D. melanogaster*, *wg*, *Notch*, *Dl*, *ser*, *vg*, *sal* and *ubx* are regulated by or interact with *ap*, with important roles in wing patterning^1^,^2^,^30^. The effects of *in vivo* knockdown of *NlapA* on expression of these genes in nymphs were investigated using qRT-PCR. In both MS and BS, knockdown of *NlapA* led to decrease in *NlDl*, *Nlsal*, *Nlser*, *Nlvg* and *Nlwg* expression (Fig. 7A,C,D,F,G). However, *Nlubx* and *Nlnotch* displayed different expression patterns between MS and BS in response to *NlapA* depletion. After ds*NlapA* injection, *Nlnotch* expression was suppressed at 2 d and enhanced at 4 d in MS, but upregulated both at 2 d and 4 d in BS (Fig. 7B). Depletion of *NlapA* resulted in downregulation of *Nlubx*, both at 2 d and 4 d in MS, while *Nlubx* expression was increased at 2 d and decreased at 4 d in BS (Fig. 7E).

### Effects of ds*NlASH* injection on expression of other genes in the wing-patterning network

**Figure 8:**
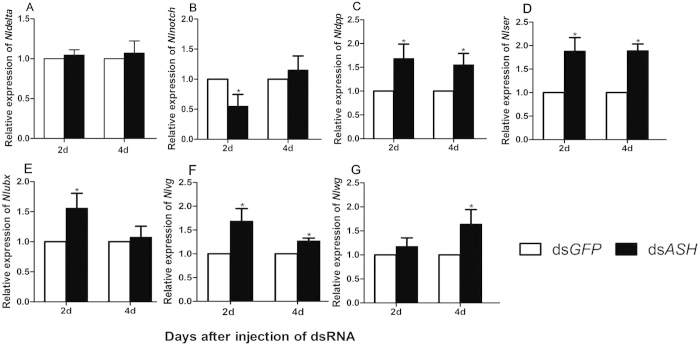
The expression levels of *Nldelta*, *Nlnotch*,*Nldpp*,*Nlser*, *Nlubx*,*Nlvg*and *Nlwg* after ds*NlASH* injection at 3rd-instar. All error bars indicate the SE of the mean, as determined from three independent replicates. The expression level was quantified relative to the value of the insects which were injected with ds*GFP*. Bars labeled asterisk differed significantly between the treatments on the same day, as determined using one way ANOVA (*P* < 0.05). BPH *actin1* was used as reference control.

In addition to loss-of-bristle forewings, knockdown of *NlASH* resulted in wing malformation. Accordingly, we examined the effects of *in vivo* knockdown of *NlASH* on expression of *wg*, *Notch*, *Dl*, *ser*, *vg*, *decapentaplegic* (*dpp*) and *ubx*. Data from qRT-PCR analysis showed that after microinjection of ds*NlASH* into 3^rd^ instar nymphs, *NlDl* expression was not affected (Fig. 8A), while *Nlnotch* was downregulated (Fig. 8B), and *Nldpp*, *Nlser*, *Nlubx*, *Nlvg* and *Nlwg* were upregulated (Fig. 8C,D,E,F,G).

## Discussion

BPH is a significant insect pest of rice. The planthopper has macropterous and brachypterous morphs, and can migrate over long distances. Although several reports have focused on the effects of environmental and genetic factors on wing development and dimorphism of BPH^26^, the underlying molecular mechanisms remain unclear. In the current study, a brachypterous pure strain (BS) and macropterous near-pure strain (MS) were obtained after 38-generation selection, providing relatively good material for the study of wing morph differentiation. In *D. melanogaster* and *T. castaneum*, the dorsal selector gene, *ap*, determines the activation of Notch signaling along the dorso-ventral boundary, in turn, inducing expression of *wg*, and acts as a long-range morphogen, providing positional information along the dorso-ventral axis by triggering expression of *vg* at different thresholds^31^. Wing-patterning genes of *D. melanogaster*, including *wg*, *Notch*, *Dl*, *ser*, *vg*, *sal* and *ubx*, interact with or are directly regulated by *ap*^1^,^2^,^30^,^32^. In *ap* mutants of *D. melanogaster*, the wing is lost^6^,^7^. Here, we cloned full-length cDNA encoding *apA* from the hemipteran insect, *N. lugens*. *NlapA* is present throughout the body from the nymphal to adult stage, and expressed highly in thorax. *NlapA* displayed different expression patterns in MS and BS. The gene was expressed more highly in nymphs of MS than those of BS at 2^nd^ and 4^th^ instar, while higher expression was detected in adults of BS than those of MS at 1 d and 3 d after emergence (Fig. 1A). ds*NlapA* injection into 3^rd^ instar nymphs caused wing malformation (loss of bristles of forewing, erect or twisted wings), along with decreased expression of *NlDl*, *Nlsal*, *Nlser*, *Nlvg* and *Nlwg* in both MS and BS groups (Fig. 7A,C,D,F,G). However, *Nlubx* and *Nlnotch* responded differently to *NlapA* depletion between MS and BS (Fig. 7B,E). Knockdown of *NlapA* in nymphs of MS additionally led to decreased wing size of adults. These results clearly indicate that *NlapA* is involved in wing patterning of *N. lugens*, and higher expression in nymphs is beneficial to generate macropterous adults. Significantly higher expression of *ap1* has been reported in winged, relative to unwinged morphs at 1^st^ and 2^nd^ instar nymphs in another hemipteran insect, *A. pisum*^2^, similar to our results. As an upstream transcription factor in the wing patterning gene network, *ap* may be an active participant in wing morph differentiation. In *T. castaneum*, injection of *T*c*ap* dsRNA into the early to late last larval stage induced deletion, marginal truncation and ventralization of the dorsal appendages^31^. In the present study, injection of ds*NlapA* into 3^rd^ instar nymphs of *N. lugens* caused phenotypic defects in wings, but no other appendages of treated insects. The issue of whether proliferation in other appendages occurs earlier than the 3^rd^ instar stage and the effects of *NlapA* knockdown require further investigation.

*ASH* encodes bHLH transcription factors and induces the formation of external sensory organs that function in mechano- and chemosensory reception. In *Drosophila*, an *ASH* double-mutant lost the majority of bristles, while ectopic expression of ac and/or sc induced extra bristles^13^,^14^,^15^,^16^. We cloned the full coding sequence of an *ASH* homologue from the hemipteran insect, *N. lugens*. *NlASH* was detected throughout the body from the nymphal to adult stage, but with significantly higher expression at the nymphal stage, there was no significant difference in *NlASH* expression level between insects of MS and BS at each development stage (Fig. 1D). Moreover, *NlASH* was expressed more highly in the thorax than other parts of the body (Fig. 1E,F). Adults of *N. lugens* developed from nymphs injected with ds*NlASH* displayed the phenotype of loss-of-bristle forewings, in accordance with the function of *ac/sc* in *Drosophila*. Additionally, knockdown of *NlASH* led to twisted or erect wing in *N. lugens*, which is not reported in *Drosophila,* signifying an additional role in the wing patterning process in this case. Injection of ds*NlASH* into 3^rd^ instar nymphs resulted in increased expression of *Nldpp*, *Nlser*, *Nlubx*, *Nlvg*, *Nlwg*, decreased expression of *Nlnotch*, and no alteration in expression of *NlDl* (Fig. 8). *In vivo* knockdown of *NlapA* and *NlASH* results in similar phenotyes, why they caused different expression trends of these wing patterning genes? No available literatures about regulation of *ASH* on these wing patterning genes could be found. Actually, according to presumptive gene network involved in wing patterning of *D. melanogaster*, *ap* is a fairly upstream regulator of wing patterning genes such as *dpp*, *ser*, *vg*, *wg*, and *notch*, while *ASH* is a fairly downstream target gene of *ap*, *dpp*, *ser*, *ubx*, *vg*, and *wg*^1^,^2^. Therefore, knockdown of *NlapA* directly regulated the expression of wing patterning genes such as *dpp*, *ser*, *vg*, *wg*, and *notch*, and depletion of *NlASH* could only indirectly regulate the expression of *dpp*, *ser*, *ubx*, *vg*, and *wg* in a feedback way. This may be the reason for different expression trends of these wing patterning genes caused by *in vivo* knockdown of *NlapA* and *NlASH*.

Expression of *ASH* is regulated by many upstream modulators, including Ap. In *Drosophila*, Chip cooperates with Pannier in bridging the GATA factor, activates AS-C through enhancer binding, and provides positional information for thorax sensory bristle patterning. Within the Pannier domain of expression, Pannier and Ap compete for binding to a common Chip cofactor. Overexpression of Pannier and absence of Ap promote the development of extra dorsocentral bristles. Thus, Ap antagonizes Pannier function, and accurate stoichiometry between these three proteins is essential for both proneural prepattern and compartmentalization of the thorax^23^. Conversely, in the present study, knockdown of *NlapA* inhibited *NlASH* expression (Fig. 5A,B), and suppressed the development of wing bristles. Further research is needed to establish the factors underlying the differential mechanisms of *ap* regulation on *ac*-*sc* between *Drosophila* and *N. lugens*.

Our results showed that *NlapA* regulates the expression of *NlASH*, and *in vivo* knockdown of *NlapA* and *NlASH* results in similar phenotyes. We are yet to establish why ds*NlASH*, but not ds*NlapA*, has lethal effects on *N. lugens*. Downregulation of *NlASH* was initiated at 4 d after *in vivo* knockdown of *NlapA* via ds*NlapA* injection into nymphs. At this time-point, nymphs were at 5^th^ instar (Fig. 5A,B). However, when nymphs at 3^rd^ instar were injected with ds*NlASH*, downregulation of *NlASH* was detected as early as 2 d after injection (at this time-point, nymphs were at 4th-instar) (Fig. 2C), and caused lethality (Fig. 6C). Thus, we assumed that knockdown of *NlASH* at 4^th^ instar may be the reason for lethality. To confirm this hypothesis, we injected ds*NlASH* into nymphs at 4^th^ instar instead of 3^rd^ instar, and examined the depletion efficiency and survival of *N. lugens*. Expression of *NlASH* was significantly decreased 2 d after ds*NlASH* injection (at this time-point, nymphs were at 5^th^ instar) (Fig. 2D), but no lethality was observed (Fig. 6D). Based on these results, we propose that *NlASH* is critical at 4^th^ instar, and its depletion at this stage induces lethality. In contrast, treatment with ds*NlapA* did not result in depletion of *NlASH* at 4^th^ instar and had no lethal effects on *N. lugens*.

Gene knockdown in planthopper may be conducted via dsRNA ingestion^27^ or injection^33^. In the current study, we injected 40 nL (10 μg/μL) dsRNA into 3^rd^ instar nymphs to eliminate expression of *NlapA* and *NlASH*. Maximal depletion of *NlapA* and *NlASH* were ~79% and ~70%, respectively. RNAi efficiency was not high, compared to that for *Spodoptera litura* (95% inhibition for the aminopeptidase gene)^34^. According to previous literature, RNAi efficiency via dsRNA injection in BPH is no greater than 90%. Injection of 0.1 μg ds*NlKr-h1* and ds*NlDll* into 3^rd^ instar *N. lugens* nymphs resulted in 78% and 87% reduction in *NlKr-h1* and *NlDll* expression, respectively^28^,^29^. After injection with 50 nL (5 μg/μL) dsRNA-*calreticulin* and dsRNA-*cathepsin-B* of BPH, maximal reduction values of *calreticulin* and *cathepsin-B* were reported as 43.8% and 36.4%, respectively^33^. RNAi efficiency in these reports was similar to that in the present study. RNAi efficiency is affected by various parameters, and may be enhanced by increasing the injection volume and dsRNA concentration, which may, however, cause high mortality^33^,^35^,^36^. In this study, we selected a fairly high injection volume and dsRNA concentration (40 nL and 10 μg/μL, respectively) to eliminate expression of *NlapA* and *NlASH*. After 10 d of injection, unspecific mortality was about 30-40%, leading to the conclusion that it is not feasible to improve RNAi efficiency by increasing the dsRNA volume and concentration. Both *NlapA* and *NlASH* are critical genes for wing development, although they are expressed throughout the nymph and adult stages. since their expression levels were fairly low (only ~0.2–0.6% that of *Nlactin1*; Fig. 1), knockdown effects were not as significant as those for highly expressed genes, which may partially explain the mild RNAi efficiency observed in the present study.

## Methods

### Insects

Wild BPH populations were collected from paddy fields in Wuhan, Hubei Province, China, and raised on TN1 (Taichuang Native 1, BPH-susceptible rice variety). Predominantly brachypterous strain (BS) and macropterous strain (MS) were selected for 38 successive generations, as described by Morooka and Tojo^37^. The percentage of brachypterous form (BS) was 100%, while the macropterous form (MS) was ~80%. Insects were raised in a growth chamber under conditions of 28 ± 1°C, 14 h light:10 h dark, and 70 ± 5% relative humidity.

### cDNA cloning of *NlapA* and *NlASH*

Total RNA was extracted from nymphs of *N. lugens* using TRIzol reagent (Invitrogen, USA). Sequences of *NlapA* and *NlASH* were obtained from the transcriptome of *N. lugens* wing pads. Expressed sequence tags (EST) of *NlapA* and *NlASH* were amplified from total RNA of *N. lugens*. A rapid amplification of cDNA ends (RACE) method was used to isolate full-length cDNA of NlapA. 3’-RACE and 5’-RACE were performed using the GenRace Core Kit (Invitrogen, USA), according to the manufacturer’s instructions. The primers used for EST cloning and RACE are shown in Supplementary Table S1.

### In silico analysis

The sequence, start codon, stop codon, molecular weight, and isoelectric point of the protein were predicted using Expasy (http://web.expasy.org/)^38^. Functional domains of the protein were predicted using InterProScan (http://www.ebi.ac.uk /interpro/)^39^. The deduced amino acid sequences were aligned with Clustal W to determine sequence identity^40^. Phylogenetic trees were built based on the amino acid sequences using MEGA 6 software with 1000 bootstraps^41^.

### Synthesis of dsRNA

The dsRNA for gene knockdown of *NlapA* was located between positions 1427 and 1985 (supplementary Fig. S2). The sequence of dsRNA for gene knockdown of *NlASH* (ds*NlASH*) is shown in supplementary Fig. S4. DsRNA was synthesized using the T7 RiboMAX Express RNAi System (Promega, USA). The sequence of a T7 polymerase promoter was fused with gene-specific primers at the 5’-end, so that the resulting PCR products contained the T7 polymerase promoter site at both ends. Amplified products were purified using a QIAquickTM PCR purification kit (Qiagen, Germany) and used as the template for *in vitro* transcription. Sense and antisense strands were transcribed from the DNA template in the same reaction. DsRNAs were extracted with phenol: chloroform and precipitated with isopropanol. Precipitated dsRNA was dissolved in nuclease-free water, heated at 75 °C for 10 min, and cooled at room temperature. *pEGFP* (GenBank accession No.U76561) dsRNA (ds*GFP*) served as a negative control. The sequences of primers used to synthesize dsRNA are presented in supplementary Table S1.

### RNA interference (RNAi) using microinjection

Third instar or fourth instar nymphs (6–12 h) of *N. lugens* were collected for microinjection, which was carried out as described by Liu *et al*^33^. Before injection, a 1.2% agarose plate was prepared and placed on an ice tray. Nymphs were anesthetized with CO_2_ for 40 s at PCO_2_ = 1 mPa and placed using a small soft brush on the agarose plate with the abdomen uppermost. Approximately 400 ng ds*NlapA*, ds*NlASH* or ds*GFP* was microinjected into each nymphal conjunctive between the prothorax and mesothorax using a Nanoliter 2000 injector (WPI, USA). For each dsRNA injection, 150 nymphs were used per replicate, with a total of three replicates (i.e., 450 total insects were injected). Treated nymphs were placed in culture dishes with fresh rice seedlings to recover and transferred into glass tubes (3 cm diameter × 25 cm length) containing seven 15-day-old TN1 rice seedlings. Each tube contained five nymphs was sealed with a nylon cover. Tubes were placed in a growth chamber at 70 ± 5% RH at 28 ± 1 °C and a 14 h:10 h (light:dark) cycle for 1-10 days. Survival rates of BPHs were recorded at 2, 4, 7 and 10 days. Meanwhile, five larvae treated with dsRNA were collected randomly at 2, 4, 7 and 10 d after injection, and total RNA extracted for qRT-PCR analysis. When adults emerged, wing phenotypes were observed under a stereomicroscope (Olympus szx16, Japan) and recorded. Twisted wings were unfolded by dipping in 5μL ddH_2_O on glass slides before measurement. Wing length was measured from the base to tip under a stereomicroscope using the cellSens Dimension 1.5 (Olympus szx10, Japan).

### qRT-PCR

Total RNA was extracted using TRIzol reagent (Invitrogen, USA). DNA contamination was removed using RNase-free DNase (Ambion, USA). *Actin1* (GenBank accession No.EU179846.1) transcript was used as the internal control gene. qRT-PCR reactions were conducted on an ABI Prism 7300 (Applied Biosystems, USA) using SYBR Premix Ex Taq (Perfect Real Time; Takara Biotechnology Corporation Co. Ltd, Dalian, China), according to the manufacturer’s instructions. Independent reactions were performed in triplicate for each RNA sample, and the signal intensity of the target gene presented as the average value. Three biological replicates were set up for each treatment. The relative expression level of genes was calculated according to the method of Livak and Schmittgen^34^. Sequences of primers for qRT-PCR are presented in supplementary Table S1.

### Data analysis

All data were analyzed using the general linear model procedure. One-way analysis of variance (ANOVA) was used to evaluate gene expression. Differences between means were examined using the Least Significant Difference (LSD) test at *P* < 0.05. Survival response to dsRNA microinjection was analyzed using Repeated Measures ANOVA. Percentage values were converted to arcsine before statistical analysis.

## Author Contributions

H.X.H. conceived the work and prepared the manuscript. F.Z.L., K.Y.L., D.B.H. and J.L performed the experiments. Y.L.Z. and Y.N.F. took the photographs. J.Z. and Y.P.H. analyzed the data.

## Additional Information

**How to cite this article**: Liu, F. *et al.* *Apterous A* modulates wing size, bristle formation and patterning in *Nilaparvata lugens*. *Sci. Rep.* **5**, 10526; doi: 10.1038/srep10526 (2015).

## Supplementary Material

Supplementary Information Supplementary Figures 1-6
