# Supplementary material for: Apterous A modulates wing size, bristle formation and patterning in Nilaparvata lugens
Source: Sci Rep. 2015 May 21;5:10526. doi: 10.1038/srep10526 (PMC4440214; doi:10.1038/srep10526)
Supplement: Supplementary Information — Supplementary Figures 1-6 [file srep10526-s1.pdf]

Electronic Supplementary Information for

Apterous A modulates wing size, wing bristle formation and wing patterning in *Nilaparvata lugens*

Fangzhou Liuł1, Kaiyin Li ł1, Jie Li2, Dingbang Hu1, Jing Zhao1, Yueping He1, Yulan Zou3, Yanni Feng3 and Hongxia Hua1*

Fangzhou Liu ł and Kaiyin Li ł contributed equally to this work

1Hubei Insect Resources Utilization and Sustainable Pest Management Key Laboratory, College of Plant Science and Technology, Huazhong Agricultural University, Wuhan, China

2College of Agronomy and Plant Protection, Qingdao Agricultural University, Qingdao, China

3College of Life Science and Technology, Huazhong Agricultural University, Wuhan, China

Table of contents

Figure S1. The homeodomain alignments of Ap Proteins

Figure S2. Nucleotide and deduced amino acid sequences of *NlapA*

Figure S3. Pylogenetic analysis of *ApA* among insects.

Figure S4. Nucleotide and deduced amino acid sequences of *NlASH*.

Figure S5. Pylogenetic analysis of *ASH* among insects.

Table S1. List of primers

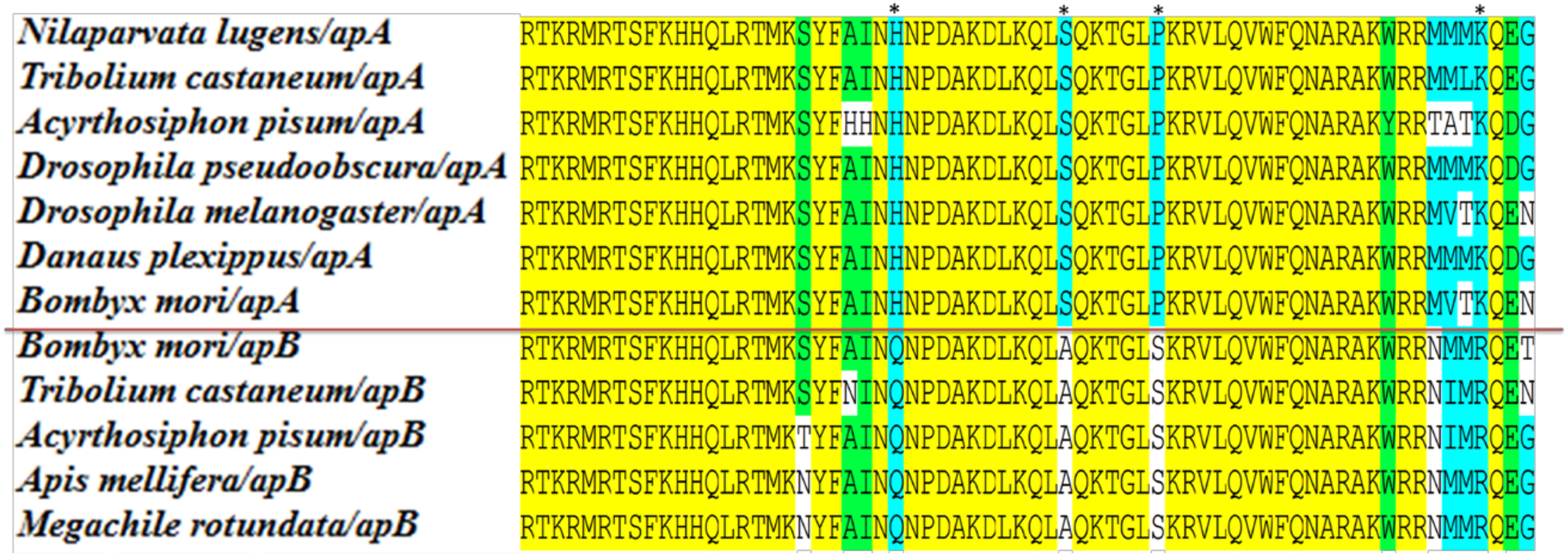

Fig. S1 The homeodomain alignments of Ap Proteins. The amino acid sequences of homeodomain of *NlapA* were aligned with *Ap* from other insects. Sequence comparisons were performed using Clustal W program. Two classes of Ap proteins (ApA and ApB) were found in insects. Asterisks indicate some amino acids unique to each class. The *Ap* coloned in the present study belong to *ApA*. The NCBI accession numbers for each of the sequences are as follows: *Tribolium castaneum* apA (NP_001139341.1), *Acyrthosiphon. pisum* apA (XP_001946004.2), *Drosophila pseudoobscura* apA (XP_004444427.1), *Drosophila melanogaster* apA (NP_724428.1), *Danaus plexippus* apA (EHJ74086.1), *Bombyx. mori* apA (BAK19079.1), *Bombyx. mori* apB (BAK19078.1), *Tribolium. castaneum* apB (FJ647812.1), *Acyrthosiphon. pisum* apB (XP_001949543), *Apis Mellifera* apB (XP_003250891.1), *Megachile rotundata* apB (XP_003700999.1).

1 CAGCGGTTGGCTAACCTGTCTACAGAAACGAAATTCGGCTTCGGGCCAACC

52 CCACTAATGGGAGTTTATGAGCTCCTTGCTGACTCTAACGGGATGCACTGG

18 M G V Y E L L A D S N G M H W

103 CAGCAGCAGCAACAGGAGCGTCCCCAGATGCTAGCGGCCGCGTACGACCCG

35 Q Q Q Q Q E R P Q M L A A A Y D P

154 TCGCGCGACCTGTCGCCCAACCTGCCAACCACATGTCACCTGGACACGCCG

52 S R D L S P N L P T T C H L D T P

205 CCGGTCGCACCGCCATGCTTCAAGACAGAAACCTCCACATCGTCCGAGTGC

69 P V A P P C F K T E T S T S S E C

LIM1

256 CACGACTGTCAATCGCCCTCGACTCCCCCCGAGCACCACCAGTCGCAGGGG

86 H D C Q S P S T P P E H H Q S Q G

307 GGCCCCACAGGGGGTCCAGGGGGCGACGAGGAGCTAGTCGTGTGTGCGGGG

103 G P T G G P G G D E E L V V C A G

358 TGCGGTGGTCGCATATGCGACCGATACTACCTGTATGCGGTCGACCGCAGA

120 C G G R I C D R Y Y L Y A V D R R

409 TGGCATGCGGCCTGTCTACAGTGTTCCCAGTGTCGTAGGGCCCTGGACAAG

137 W H A A C L Q C S Q C R R A L D K

LIM2

460 GAGGTCACCTGTTTCGCACGTGATGGCAGTATCTTCTGCAAAAAGGACTAC

154 E V T C F A R D G S I F C K K D Y

511 TATAGGTTATTCGGCGCACGTCGCTGCGGCCGATGTCAGGCGTCCATTCTG

171 Y R L F G A R R C G R C Q A S I L

562 TCATCAGAGCTTGTGATGCGAGCCCGCGACCGTGTTTTCCACGTGAACTGC

188 S S E L V M R A R D R V F H V N C

613 TTCAGCTGTTCCGTCTGCTCAGCTGTGCTCACCAAGGGGGACCATTTCGGC

205 F S C S V C S A V L T K G D H F G

664 ATGCGGGACGGGGCCGTCTTCTGCCGCCTCCACTACCACGAGCTGCCTTCG

222 M R D G A V F C R L H Y H E L P S

715 CCGATGGGGGGCGGCTCCACCCCCGACTACCACTACCACCCCCATCCGCAC

239 P M G G G S T P D Y H Y H P H P H

766 CCGCCTCCGATGCCCTCGCCGGTCAGTGCCGCCCCCGCCGACCCGGTCAAG

256 P P P M P S P V S A A P A D P V K

817 GTAGCCGGGTCCTTCTTCAACGGAGTGCCCACCCCAAGGCAGAAGGGGCGG

273 V A G S F F N G V P T P R Q K G R

868 CCAAGGAAGCGCAAGCCCAAAGATCTCGAAGGCATGACCGCTAATCTCGAC

Homeodomain

290 P R K R K P K D L E G M T A N L D

919 CTAAACGCGGACGCCTACCTGGAGGTGGCGTTTGGCCCCGGGACCCCTGGC

307 L N A D A Y L E V A F G P G T P G

970 ATGGGGGGCGCAAACGGCAGTCACCATCAGCGCACAAAGCGCATGCGAACA

324 M G G A N G S H H Q R T K R M R T

1021 TCGTTCAAGCACCATCAACTGCGCACCATGAAGTCATACTTCGCCATCAAC

341 S F K H H Q L R T M K S Y F A I N

1072 CACAACCCTGACGCCAAGGACCTCAAACAGCTCTCACAAAAGACTGGTCTG

358 H N P D A K D L K Q L S Q K T G L

1123 CCCAAGAGGGTACTACAGGTATGGTTCCAGAACGCGAGAGCAAAATGGCGA

375 P K R V L Q V W F Q N A R A K W R

1174 CGGATGATGATGAAACAGGAGGGTGGTGGCAAGGGTGGATCGCTGGACAAA

392 R M M M K Q E G G G K G G S L D K

1225 TGTGGGGATGGCTCCAGCACATCGCTGGAGGCTGCCTATCATCTTCCCTCT

409 C G D G S S T S L E A A Y H L P S

1276 CACAGTCCGCAGCATTATCTCAGTTCATCCCCACTAGAATGCTCATCGTGA

426 H S P Q H Y L S S S P L E C S S

1327 AATCCACTCTTCCTCAATTCCTTGTAACGGACATTTTTGTGACGAGGAAGT

1378 GCTCCAATTGAGCCAACAACAAACTAAAACTAAGTATTCAATCACTCACTA

1429 ACCGTCAAAAACCTCACGTTTTGATGTTAAACGTCAAGGATTTTTGATGAT

1480 GATTATCACTGCTTGATGGTGACGTTTATCATAGTTTGAATATGATTATCA

1531 CTGGTAGATGATGATTGTCACAGGACACTGGTTGATGATAATTTTTATCAT

1582 AGTTTGATGATGATTATCACTGGTAGATGATGATTGTCACTGGACACTGGT

1633 TGATGATGATTATCACTGGTTTATGATGATTATCATAGGTTGATAATCATC

1684 ACGGATTGATGATGATTATCACTGGTTGATGATGACGTTCATCACAGTTTG

1735 ATTATTATTATCACTGACTGATAATATTTATCACCAGATTGGTCATCAACA

1786 TCACTCGTTGATGGTCATTAAAGCTTGATAAAAGTCATCATGGGTTGATGA

1837 TTATCACAAGTTGATGATAAACATCAAGGGTTGATGATTTTTATCTCTTAA

1888 TCGTGAACATCTTTGGTTGATGATGATCATCATCAATGTGATTAAAATTAA

1939 TTAATCAATGGTTGATGATGATTATCACTGGTTGGTCGTGAATATCGTTGG

1990 TTGATGATGATTATCATTGGTTGATCGTGATTATCACGGTTCATGTTGATC

2041 TTCACAGGTTGTTAATCATAACAAGTTGATGAAACTCTCTACCAGTTGATA

2092 ATCATCAAGGGTTGATGACAATCATCGAAGGTTGATGATAATCATCAGGGG

2143 TTGATGATAATCATCACTGTTGTGTTGTGAGGGTGCAAAAAGTAACTGTGA

2194 ATCAATCTAGTAAAACCATGAAAAACTCGTTGGTAGATAGCATAATTTATT

2245 GAGGAATAAATAGTGAACAATGTAATTTAGACTTCTTATTCAATCCGTTGT

2296 GGAATTTGGTAGAGTTTGTTGGAGATGAGTGAGGATGGAATAGTGATAAAT

2347 GAAATTTAGACTACCTAATTAATCTGTGAAATCAATGTGTGTATATCTGTG

2398 AACAGAACGGAACAAGAGCCTACAAATATAATAAATTATATTGATTTATTA

2449 AATTTATATTGGAAATCAACGGGTGAGCTTATGTAATTCTACTCAACTCTG

2500 GAAGCTGTGGCTAATGCTTTGTTAAAAGAGAAAGATAACGAATCAATTAAC

2551 ATTGTGGAAAGAGACAAAATATCAGTACGGTAAACATTTTTAAAGCGAGAT

2602 ATTATTAGTTTTTAATAAACAGTTAATCATTATTTTCGAGTTCTAAAGAGA

2653 AGTTATTGAAAATTTGAAGTGATTTCCTTTCAAACTATGATAATCTTTTAG

2704 AAGATTTTCCAAAACAATTTTTAAATTCTAGAAATTATTCCCCAATAGGTA

2755 TCAGTGTTAAAAAATATTTAAAATGTTACAAACAAAACAAAAAACAGTATA

2806 CACGTTCAGAACGTTGATAAAAATGGAATATTATTGTAATAAATAAAAAAA

2857 GAATCATGATGTTTTAGGACAAAAATGATTGTTTTCTTCAAATACAAGACT

2908 GATTCAGTAAGCTGAAGAAAATATGTATGATAGATTTAAAATTTATTCAAA

2959 ACGTTTCTAAAACTAATTTGAAACCTAGAAAAAAAAAAAAAAAAAA

Fig. S2 Nucleotide and deduced amino acid sequences of *NlapA*. The start codon was labeled with an asterisk, and the stop codon was labeled with a triangle. Two LIM domains (*LIM1* and *LIM2*) are boxed, the homeodomain is underlined. The corresponding sequences of ds*NlapA* were shaded in grey.

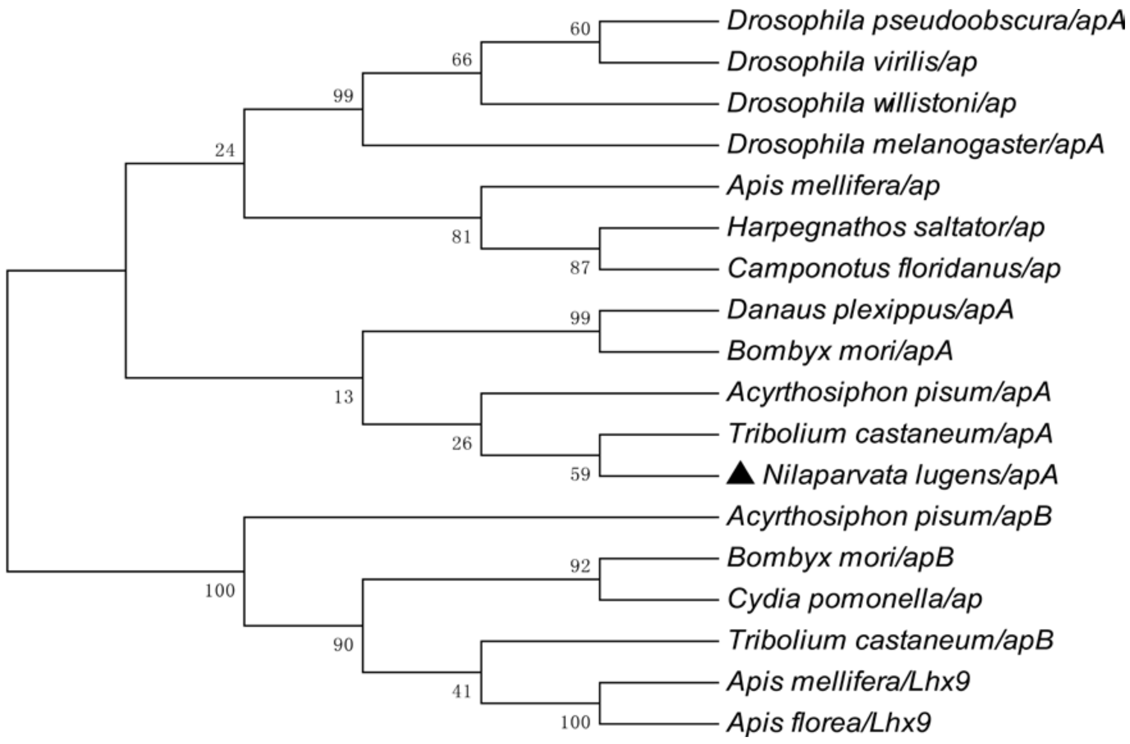

Fig. S3 Pylogenetic analysis of *ApA* among insects. Accession numbers: *Drosophila pseudoobscura* apA (XP_004444427.1), *Drosophila virilis* ap (XP_002059906.1), *Drosophila willistoni* ap (XP_002061119.1), *Drosophila melanogaster* apA (NP_724428.1), *Apis mellifera* ap (XP_392622.4), *Harpegnathos saltator* ap (EFN77730.1), *Camponotus floridanus* ap (EFN60497.1), *Danaus plexippus* apA (EHJ74086.1), *Bombyx mori* apA (BAK19079.1), *Acyrthosiphon pisum* apA (XP_001946004.2), *Tribolium castaneum* apA (NP_001139341.1), *Nilaparvata lugens* apA (KC978728), *Acyrthosiphon pisum* apB (XP_001949543.1), *Bombyx mori* apB (BAK19078.1), *Cydia pomonella* ap (AFD93370.1), *Tribolium castaneum* apB (FJ647812.1), *Apis mellifera* Lhx9 (XP_003250891.1), *Apis florea* Lhx9 (XP_003697250.1).

```
1     ATGGGTGGAGGAAGATGTGCCGGTGGCTGGAGAGGCGCCACTAGTGGGGTGGTGGGGGGT
      ★
1     M  G  G  G  R  C  A  G  G  W  R  G  A  T  S  G  V  V  G  G
61    GTCGCGTGCCGAGCGAGGCCACGCGCCGAGCGGCGCGCGCTGTCAGTGGAGGGGGCAGCT
21    V  A  C  R  A  R  P  R  A  E  R  R  A  L  S  V  E  G  A  A
121   ATAAAAAGGGCCGGAAACTGGCAGCGCGCGTCATTCGCACTCGAGATGGAGACACATGAG
41    I  K  R  A  G  N  W  Q  R  A  S  F  A  L  E  M  E  T  H  E
181   GTTCGTTGCAAGCGGAGAATCAGTTTCGCGTACGGCGGACAGCAGACGGCGTCGGTAGCG
61    V  R  C  K  R  R  I  S  F  A  Y  G  G  Q  Q  T  A  S  V  A
241   CGTCGCAACGCACGTGAGCGCAACCGCGTCAAACAGGTGAACAACGGATTCGCGACGCTA
81    R  R  N  A  R  E  R  N  R  V  K  Q  V  N  N  G  F  A  T  L
301   CGGGCGCACATCCCCGTGTCGGTGACGGCGGCCCTTGGCGGACAAACCCAGCGGCCCGCA
101   R  A  H  I  P  V  S  V  T  A  A  L  G  G  Q  T  Q  R  P  A
                              HLH
361   CCCGGCAGTGCCGCCTCCAAGAAGCTCAGCAAAGTCGAGACCCTGAGGATGGCAGTCGAG
121   [P  G  S  A  A  S  K  K  L  S  K  V  E  T  L  R  M  A  V  E]
421   TATATCCGCTCCCTCCAACAACTCCTCGACGGACAAGTCGTATCGCCCCCACCCTCCTCC
141   [Y  I  R  S  L  Q  Q  L  L] D  G  Q  V  V  S  P  P  P  S  S
481   TCGTCACCCGTCAGTATGGCGTCGCCGCACTGTTCGGAGGCGAGCAGTTCGCCGCCTCCG
161   S  S  P  V  S  M  A  S  P  H  C  S  E  A  S  S  S  P  P  P
541   AGCTCCTACAACTCGGACTCCGCCCCCGGACAGACGTCTGTGCCCTCCTACAACCACTAC
181   S  S  Y  N  S  D  S  A  P  G  Q  T  S  V  P  S  Y  N  H  Y
601   CACCACGTTCAGCCCATGTCCCCCGAGGATGAGGAATTGCTCGACGTTATTTCATGGTGG
201   H  H  V  Q  P  M  S  P  E  D  E  E  L  L  D  V  I  S  W  W
661   CAGCAGACGCAATGACCTTCAAGGAACGGGGGCGACTCTCATTCCCTTCCACTGATCTCA
221   Q  Q  T  Q  ▲
```

Fig. S4 Nucleotide and deduced amino acid sequences of *NlASH*. The start codon was labeled with an asterisk, and the stop codon was labeled with a triangle. The HLH domain are boxed. The corresponding sequences of ds*NlASH* were underlined.

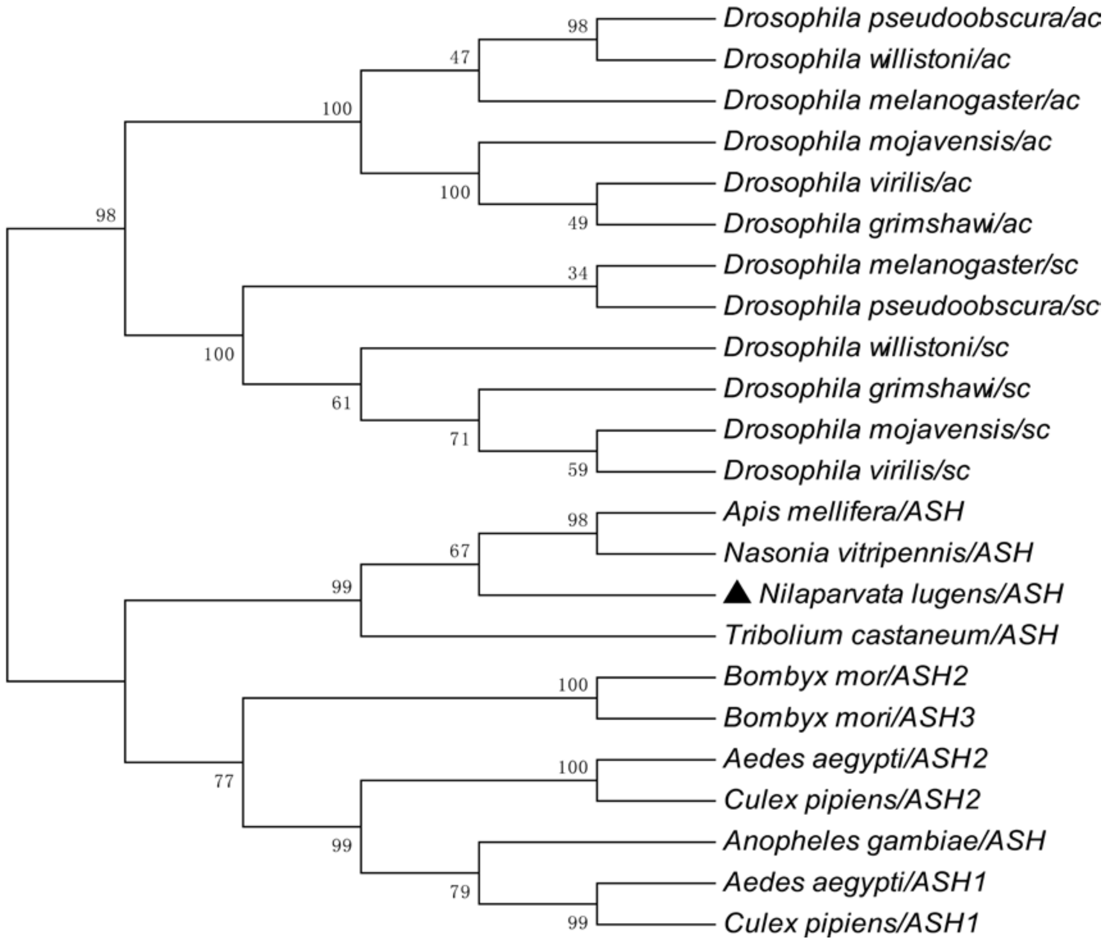

Fig. S5 Pylogenetic analysis of ASH among insects. Accession numbers: *Drosophila pseudoobscura* ac (XP_001354784.2), *Drosophila willistoni* ac (XP_002071187.1), *Drosophila melanogaster* ac (NP _476824.1) , *Drosophila mojavensis* ac (XP_002011619.1), *Drosophila virilis* ac (XP_002058253.1), *Drosophila grimshawi* ac (XP_001992198.1), *Drosophila melanogaster* sc (NP_ 476803.1), *Drosophila pseudoobscura* sc (XP_002134089.1), *Drosophila willistoni* sc (XP_002071186.1), *Drosophila grimshawi* sc (XP_001992197.1), *Drosophila mojavensis* sc (XP_002011618.1), *Drosophila virilis* sc (XP_002058251.1), *Apis mellifera* ASH (NW_001252982), *Nasonia vitripennis* ASH(XP _003426998.1), *Nilaparvata Lugens* ASH (KM_244736), *Tribolium castaneum* ASH(NP_ 001034537.1), *Bombyx mori* ASH2 (NP_001098692.1), *Bombyx mori* ASH3 (NP_001098694.1), *Aedes aegypti* ASH2 (XP_001659152.1), *Culex pipiens* ASH2 (XP_001849852.1), *Anopheles gambiae* ASH (AAK_97461.1) , *Aedes aegypti* ASH1 (XP_001659150.1), *Culex pipiens* ASH1 (XP_001869041.

Table S1. List of primers

Gene (Accession No.)	Name of primer	Name of primer
NlapA (KC978728)	primers for EST cloning	F: TATAGGTTATTCGGCGCACGT
		R: GATTTCACGATGAGCATTCTA
	primers for 5'-RACE	GSP1: ACGGTCGCGGGCTCGCATCACAA
		GSP2: GCATCACAAGCTCTGATGACAGA
	Primers for 3'-RACE	Out primer: ATGTCAGGCGTCCATTCTGTCAT
		Inner primer: TCTGTCATCAGAGCTTGTGATGC
	Primers for qRT-PCR	F: CCAACCTGCCAACCACATG
		R: AGTCGAGGGCGATTGACAGT
	Primers for ds*NLapA* synthesis	Sense: GGATCCTAATACGACTCACTATAGGAGTGTTCCCAGTGTCGTAGG
		Antisense: GGATCCTAATACGACTCACTATAGGCGCGTTTAGGTCGAGATTAG
NlASH (KM244736)	primers for EST cloning	F: AGATGTGCCGGTGGCTGGAG
		R: GAATACCGAAATAACATAAACG
	Primers for qRT-PCR	F: GAGATGGAGACACATGAGGTT
		R: GAATCCGTTGTTCACCTGTTT
	Primers for ds*NLASH* synthesis	Sense: TAATACGACTCACTATAGGCCTCGTCACCCGTCAGTATGG
		Antisense: TAATACGACTCACTATAGGTGGGCTGAACGTGGTGGTAGT
pEGFP (U76561)	Primers for ds*GFP* synthesis	Sense: GGATCCTAATACGACTCACTATAGGGTAAACGGCCACAAGTTCAG
		Antisense: GGATCCTAATACGACTCACTATAGGTCGGCCATGATATAGACGTT
Actin1 (EU179846.1)	Primers for qRT-PCR	F: CCAACCGTGAGAAGATGACC
		R: GATGTCACGCACGATTTCAC

Table S1. List of primers (continued)

Gene (Accession No.)	Name of primer	Name of primer
Nlvg (KM264381)	Primers for qRT-PCR	F: CACCTGAAACAACTGGAGACG
		R: AGAGTGCCCTGGTGAAGTGTT
Nlubx (KM264379)	Primers for qRT-PCR	F: CTTTGAACAGTCGGGCTTCT
		R: GTGGTGGTGTTGACTGCTTG
Nlwg (KM264384)	Primers for qRT-PCR	F: CACTGGTGCTTCTCACGCTT
		R: GGGTAGCAGGTTATTCGGCT
NlDl （KP196804）	Primers for qRT-PCR	F:CTCCCACTCCTACACCCAACACAA
		R:TGCTTCTGAGACCTGCTCCTGT
Nlser （KP196803）	Primers for qRT-PCR	F:GTCTGGCAAGTGGCGTTGTAGT
		R:CGTCCTACTCTGGCATCATCCT
Nlnotch （KP196805）	Primers for qRT-PCR	F:GGTGCAGGTTGGAATGTGGTTGAT
		R:TCCGACAGACAGAGCGTGTGTT
Nldpp (KM264380)	Primers for qRT-PCR	F: CGGCTATGACGCGTACTACTG
		R: CACAGAGTGCACGAGTGTCTG
Nlsal (KM264383)	Primers for qRT-PCR	F: TACTGTGGGAAGGTGTTTGG
		R: GGTGGGTGATACTTGTCCAG
